# The Long-Term Safety and Quality of Life Effects of Oats in Dermatitis Herpetiformis

**DOI:** 10.3390/nu12041060

**Published:** 2020-04-11

**Authors:** Anna Alakoski, Kaisa Hervonen, Eriika Mansikka, Timo Reunala, Katri Kaukinen, Laura Kivelä, Pilvi Laurikka, Heini Huhtala, Kalle Kurppa, Teea Salmi

**Affiliations:** 1 Department of Dermatology, Tampere University Hospital, 33521 Tampere, Finland; anna.alakoski@pshp.fi (A.A.); kaisa.hervonen@tuni.fi (K.H.); timo.reunala@tuni.fi (T.R.); 2 Celiac Disease Research Center, Faculty of Medicine and Health Technology, Tampere University, 33014 Tampere, Finland; eriika.mansikka@terveystalo.com (E.M.); katri.kaukinen@tuni.fi (K.K.); laura.kivela@tuni.fi (L.K.); pilvi.laurikka@tuni.fi (P.L.); 3 Department of Internal Medicine, Tampere University Hospital, 33521 Tampere, Finland; 4 Faculty of Social Sciences, Tampere University, 33014 Tampere, Finland; heini.huhtala@tuni.fi; 5 Department of Paediatrics, Tampere University Hospital, 33521 Tampere, Finland; kalle.kurppa@tuni.fi; 6 Center for Child Health Research, Tampere University; 33521 Tampere, Finland; 7 Department of Pediatrics, Seinäjoki Central Hospital and the University Consortium of Seinäjoki, 60220 Seinäjoki, Finland

**Keywords:** dermatitis herpetiformis, coeliac disease, gluten-free diet, oats, quality of life, complications, follow-up

## Abstract

The treatment of choice for dermatitis herpetiformis (DH), a cutaneous manifestation of coeliac disease, is a life-long gluten-free diet (GFD). In a GFD, wheat, rye and barley should be strictly avoided, but the role of oats is more controversial. This study aimed to investigate the safety and long-term quality of life and health effects of oat consumption in 312 long-term treated DH patients. Baseline data were gathered from patient records and follow-up data from questionnaires or interviews, and validated questionnaires were used to assess quality of life. We found that altogether 256 patients (82%) were consuming oats as part of their GFD at the follow-up. Long-term follow-up data showed that there were no differences in the presence of long-term illnesses, coeliac disease complications or the usage of medication between those consuming and not consuming oats. However, oat consumers had a better quality of life and reported ongoing gastrointestinal symptoms less frequently (4% vs 19%, *p* = 0.004) at the follow-up than those not consuming oats. The study established that oats are safe for DH patients and in the long-term seem to improve the quality of life of DH patients.

## 1. Introduction

Dermatitis herpetiformis (DH), a blistering and itching autoimmune skin disease, is considered a cutaneous manifestation of coeliac disease [1]. In DH and coeliac disease, the disorder is induced by gluten in genetically susceptible individuals. Although patients with DH infrequently suffer from prominent gastrointestinal symptoms, the majority of them have classical coeliac-type villous atrophy in the small intestine at diagnosis [2,3]. Moreover, targeted antibody response in the serum to tissue transglutaminase (TG2) is characteristic of both coeliac disease and DH [4,5]. Pathognomonic for DH, however, is the presence of granular IgA deposits in the dermis targeted against epidermal transglutaminase (TG3), not TG2 [6].

The treatment of choice for DH and coeliac disease is a lifelong gluten-free diet (GFD). Strict adherence to a GFD eventually leads to the disappearance of clinical symptoms and improvement of the small-intestinal villous architecture [7,8,9,10], as well as the normalisation of serum TG2 antibodies [11,12]. Additional advantages of a GFD include a decreased risk for lymphoproliferative malignancies [13,14] and bone fractures [15], and increased quality of life [16,17].

There are, however, some disadvantages to a GFD, as it is restrictive and expensive to maintain [18], and it may lead to suboptimal nutrition, such as high sugar and low fibre and mineral intake [19]. There is also some confusion about the contents of the GFD recommended to coeliac disease and DH patients. Generally, there is worldwide consensus on the toxicity of wheat, rye and barley in coeliac disease and DH. However, the role of oats—which have a different storage protein composition from wheat, rye and barley—in a GFD remains controversial [20]. In coeliac disease, the majority of performed studies with short- and long-term follow-ups have demonstrated that the consumption of oats is safe [21,22,23,24]. However, gastrointestinal symptoms, intestinal inflammation and even small-intestinal villous atrophy have also been associated with an oat-containing GFD in coeliac disease [25,26,27]. Moreover, only scarce knowledge exists concerning the safety of oat consumption in DH. According to previously performed DH oat challenge studies with a follow-up of up to six months, oats seem to be safe for DH patients [28,29]. However, the long-term safety of oat consumption in DH remains uncertain, and further evidence is called for especially since oats are an essential source of vitamins, minerals, soluble fibre and polyunsaturated fatty acids. Moreover, oats are known to have multiple health advantages, such as positive effects on blood glucose and cholesterol levels and the maintenance of normal body weight and blood pressure [30,31]. Importantly, in coeliac disease and DH, the consumption of oats diversifies the diet of the patients [32].

In Finland, uncontaminated oats have been accepted as a constituent of a GFD for more than two decades [32], and nowadays products containing uncontaminated oats are widely available in grocery stores. Furthermore, DH prevalence and DH patients’ adherence to a GFD are known to be exceptionally high in Finland [33,34]. This offered us an excellent opportunity to evaluate for the first time the long-term safety and quality of life effects of oat consumption in DH.

## 2. Materials and Methods

### 2.1. Patients and Study Design

The study comprised 312 DH patients, who were gathered from two different cohorts: 224 patients were collected in 2016 from DH patients diagnosed at the Department of Dermatology, Tampere University Hospital between 1970 and 2014 (cohort 1); and the remaining 88 DH patients were recruited between 2006 and 2010 by a nationwide search via a newspaper advertisement and with the help of national and local coeliac disease societies (cohort 2).

For each patient, the DH diagnosis was based on the typical clinical picture and the demonstration of granular IgA deposits in the papillary dermis with direct immunofluorescence examination. After the diagnosis, all DH patients were routinely recommended to undergo gastroscopy with small bowel biopsies. After the gastroscopy, patients were advised to adhere to a strict, life-long GFD by a dermatologist, and dietary advice was given by a dietician. In those patients with severe skin symptoms, dapsone medication was also initiated.

DH patients diagnosed at any age were included in the study and serological response to GFD was evident in all 79 DH patients with available follow-up data. Five DH patients not adherent to a GFD, one patient without follow-up data on oat consumption, and seven patients without biopsy proven DH diagnosis were excluded from the final analysis. In addition, 11 patients were excluded from cohort 2 as they were already included in cohort 1.

Follow-up data from cohort 1 study patients were gathered using a special study questionnaire designed for DH patients [35] and from cohort 2 study patients via interviews conducted by an experienced physician or study nurse.

The Regional Ethics Committee of Tampere University approved the study protocol and usage of register-based data. All subjects gave their written informed consent.

### 2.2. Clinical and Dietary Information

Data on demographic characteristics, the presence and severity of DH- and coeliac disease-related clinical symptoms, small bowel mucosal findings and the results of serum coeliac autoantibodies at diagnosis were gathered from the patient records. The small bowel mucosal histological analysis, interpreted by an experienced pathologist, was available from 243 patients, and the result was graded as subtotal or total villous atrophy, partial villous atrophy or normal mucosa, as previously described [2]. The serum coeliac disease autoantibody tests used were reticulin antibody (ARA), endomysium antibody (EmA) or TG2 antibody tests, depending on the time of the testing. In the ARA and EmA tests, titers 1:≥5 were considered positive, and in the TG2 antibody tests, the reference value was 20 or 5, depending on whether an INOVA (INOVA Diagnostics, San Diego, CA, USA) or Celikey (Celikey Pharmacia, Uppsala, Sweden) test was used. The ARA, EmA and TG2 antibody tests are all directed against TG2 [36], and they are collectively referred to as serum coeliac autoantibodies in this article. Data on the duration and severity of skin symptoms at diagnosis and initiation of dapsone were only gathered from cohort 1 patients. The skin symptoms were graded as mild, moderate or severe by one dermatologist, and the grading was based on the presence of a few, several or many blisters, macular eruptions and erosions.

At the follow-up, data on oat consumption and the duration, adherence and possible lapses of the GFD were gathered. GFD adherence was interpreted as no dietary lapses, dietary lapses less than once a month or dietary lapses once a month or more often. Data on chronic illnesses, coeliac disease complications, and the family history of coeliac disease or DH were recorded at the follow-up. Further, previous bone fractures and malignancies were recorded, but excessive trauma fractures and non-melanoma skin cancers were excluded from further analysis. Specific questions about the frequency of oat consumption (classified as twice a week or more often, less than two times a week or no use), the presence of ongoing skin and gastrointestinal symptoms, the current usage of dapsone and other physician-prescribed and over-the-counter (OTC) medications, physical exercise, smoking, number of children born and current weight and height were asked in the study questionnaire gathered from cohort 1 patients diagnosed, but not recorded during the study interviews of cohort 2 patients.

### 2.3. Questionnaires

The study questionnaire and interviews used in the current study were structured and included multiple choice and open questions designed by physicians with expertise in coeliac disease and DH. In addition, all participants filled the validated gastrointestinal symptom and quality of life questionnaires (see below in more detail).

#### Validated Questionnaires

The presence of gastrointestinal symptoms and quality of life at the time of the follow-up were gathered with the Gastrointestinal Symptom Rating Scale (GSRS) [37], Psychological General Well-Being (PGWB) [38] and Dermatologic Life Quality Index (DLQI) [39] questionnaires. GSRS and PGWB are validated questionnaires that have been broadly used in previous coeliac disease studies [21,25,40]. DLQI is a validated questionnaire commonly used to assess quality of life associated with any type of dermatological disease [41].

GSRS is a 15-item questionnaire that evaluates the severity and occurrence of gastrointestinal symptoms in five sub-categories: diarrhoea, indigestion, constipation, abdominal pain and reflux. It uses a seven-point Likert scale for each question. Sub-scores for each of the five categories are calculated as the average of three relevant items and the total score is calculated as the average of all 15 items. The total score ranges from 1 to 7, and a higher score means more severe symptoms.

PGWB is a 22-item questionnaire that estimates self-perceived health-related well-being and distress. It includes six dimensions: anxiety, depressed mood, positive well-being, self-control, vitality and general health. The total score ranges from 22 to 132. Higher scores indicate better quality of life.

The DLQI is a 10-item quality of life implement. The questionnaire includes six different divisions: symptoms and feelings, daily activities, leisure, work and school, personal relationships, and treatment. The scores of all ten questions are calculated together, and the total score varies from a minimum of 0 to a maximum of 30. A higher score indicates poorer quality of life. dermatology-specific.

### 2.4. Statistics

The participants were divided into two groups depending on the consumption of oats and then compared. Categorical variables are presented as percentages and continuous variables as medians with ranges or quartiles (Q_1_–Q_3_). Cross-tabulation with a two-sided chi-squared test was used to compare the categorial variables, and the Mann–Whitney *U* test was used to measure the difference between continuous variables. All statistical analyses were made using SPSS version 25.0. Statistical significance was set at *p* < 0.05.

## 3. Results

### 3.1. Baseline data of DH Patients on a Gluten-Free Diet with and without Oats

The median age of the 312 DH patients was 37 years and 49% were females. There were no differences in the gender, age, year of the DH diagnosis, or duration of skin symptoms or gastrointestinal symptoms before being diagnosed between DH patients using and not using oats. There was a trend towards patients not using oats having more severe skin symptoms at diagnosis, but the difference did not reach statistical significance (*p* = 0.076). The severity of villous atrophy and the percentage of serum coeliac antibody-positive patients at diagnosis did not differ between the study groups (Table 1). After being diagnosed, dapsone treatment was initiated in 129 (73%) out of the 176 DH patients using oats and in 30 (86%) out of the 35 DH patients not using oats (*p* = 0.119).

### 3.2. Follow-Up Data of DH Patients on a Gluten-Free Diet with or without Oats

In all, 256 patients (82%) were currently using oats, and oat consumption did not differ significantly between cohort 1 and cohort 2 (84% vs 79%, *p* = 0.168). Out of all oat consumers, 72% were consuming oat-based products two times a week or more and 28% were consuming oat-based products less than two times a week. There were no differences in the median duration of the GFD, adherence to the GFD or the prevalence of current skin symptoms between the two study groups (Table 2). However, DH patients not consuming oats reported suffering from gastrointestinal symptoms significantly more often (19% vs 4%, *p* = 0.004), and they needed dapsone treatment more frequently (14% vs 4%, *p* = 0.040) at the follow-up compared to the oat consumers (Table 2). The number of long-term illnesses, previous bone fractures and malignancies, and regularly used prescription or OTC medications were similar in the two study groups (Table 2). Likewise, there were no differences between the study groups in the amount of physical exercise taken or BMI, but patients not using oats were more frequently current smokers (*p* = 0.032) (Table 2).

In the GSRS questionnaire, the total scores did not differ significantly between the two study groups. In the sub-score analysis the diarrhoea sub-score was significantly higher among those not consuming oats (*p* = 0.045, Table 3). In the PGWB questionnaire, general health as well as vitality were significantly higher in the oat-consuming group (*p* = 0.020 and *p* = 0.025, respectively), but the total score and other sub-scores did not differ between the study groups (Table 3). Likewise, in the DLQI questionnaire, dermatological quality of life scores were higher among oat consumers compared to those not consuming oats (*p* = 0.028) (Table 3).

## 4. Discussion

The current study showed that as many as 82% of Finnish DH patients on a GFD consume oats regularly. Comparisons with DH patients not using oats established that the long-term consumption of oats does not cause additional skin symptoms or morbidity, as the incidence of DH-related complications and other long-term illnesses was shown to be comparable between DH patients consuming and not consuming oats as part of their GFD. Importantly, the usage of oats by DH patients on a GFD was associated with fewer gastrointestinal symptoms and better quality of life.

To our knowledge, there are only three previous studies concerning oat consumption in DH, two of which are oat challenges. Hardman et al. [29] performed a three-month oat challenge study involving 10 GFD-treated DH patients, and they observed no rash, serum coeliac autoantibodies, or damage to the small bowel mucosa. In a previous Finnish study [28], 11 GFD-treated DH patients were challenged daily with 50g of oats for six months, while a control group of 11 DH patients continued their conventional GFD without oats. The oat challenge did not cause any changes in the villous architecture or coeliac serology, but two challenged patients experienced a transient rash and one patient withdrew because of a more persistent, albeit mild, rash. Although the numbers of patients in the challenge studies were small and the follow-up times short, the results are in line with the current study demonstrating the safety of long-term oat consumption by DH patients. Moreover, a previous questionnaire study from Finland [32] investigated oat consumption in GFD-treated coeliac disease and DH patients. It demonstrated that several DH patients (19%) had stopped using oats mainly because of cutaneous symptoms. However, it remained unknown whether the skin symptoms were oat-related, nor was it known how long the patients had been using oats before cessation. As previously suggested [28], it is possible that some DH patients may experience mild and transient skin symptoms after the introduction of oats to the GFD. However, this seems to be of minor importance in the long run, since as many as 82% of the DH patients in the present long-term study continued to consume oats in their GFD.

Compared to DH, there are considerably more studies addressing oat consumption in coeliac disease. According to a recent meta-analysis [42], the vast majority of the performed studies have demonstrated that oat consumption does not cause short- or long-term harm to coeliac disease patients. In most of the studies, which feature oat consumption lasting up to ten years, no changes in duodenal villous architecture, coeliac disease serology or gastrointestinal symptoms were detected among those using oats [21,22,24]. However, since there are also a few reports demonstrating increased gastrointestinal complaints, intraepithelial lymphocytosis and even damage of the small bowel mucosal villi in coeliac disease patients consuming oats [25,26], this issue is not totally undisputable. Hence, in a recent review of GFD treatment in coeliac disease [20], it is stated that the long-term risks of consuming oats in coeliac disease remain unknown and further studies are warranted.

The present study also investigated the factors predicting oat consumption in DH. Other than the detected – albeit not statistically significant – difference in the severity of skin symptoms, the baseline characteristics among those using and not using oats did not differ. Intriguingly, at the follow-up, DH patients not using oats reported suffering from gastrointestinal symptoms more often than those consuming oats. Moreover, they had slightly more frequent skin symptoms and more often used dapsone treatment than the oat consumers. It is possible that the DH patients with more severe and/or persistent skin symptoms are not willing to include oats in their GFD due to the fear of worsening their symptoms. At the follow-up, the DH study patients did not differ in terms of BMI and the presence of long-term illnesses or coeliac disease complications, and the number of prescription medications was similar. These results show that oat consumption does not have negative effects on the long-term prognosis of DH.

In fact, an important finding in the present study was that the quality of life measured with the DLQI questionnaire was significantly better in patients consuming oats than in patients not consuming oats. Correspondingly, oat consumers reported significantly better general health and vitality when measured with the PGWB questionnaire. Our findings are in line with previous reports on the quality of life effects of oat use in Finnish coeliac disease patients, with the quality of life of oat consumers being even better than that of those not consuming oats [21,25]. It has been reported that coeliac disease patients find that oats diversify their GFD in many ways; patients especially appreciate the taste, ease of use, and low cost of oats [32], all of which most likely have positive effects on their quality of life.

The main strengths of the present study are the large, well-defined study group with a skin immunofluorescence biopsy-proven DH diagnosis and the long duration of follow-up (a median of over 20 years). Further strengths include the use of validated questionnaires to investigate quality of life and gastrointestinal symptoms, and the possibility to gather comprehensive follow-up data in terms of the prognosis of DH.

A limitation was that the follow-up information was based on questionnaires and interviews, which may have yielded selection and recall bias. Further, we did not ask the reasons for not consuming oats and the duration of oat consumption is not known in those using oats. Finally, duodenal biopsies and coeliac serology were not available at the follow-up. On the other hand, it has previously been shown that duodenal recovery in GFD-treated DH patients is excellent in Finland [43]

## 5. Conclusions

To conclude, we demonstrated that oats are widely used as a constituent of a GFD among Finnish DH patients. Long-term consumption of uncontaminated oats is safe and even seems to improve the quality of life of DH patients. Based on the current long-term study and earlier challenge studies, the inclusion of uncontaminated oats in the diet of DH patients is justified.

## Figures and Tables

**Table 1:** Demographic data and clinical, serological and histological findings at diagnosis in 312 dermatitis herpetiformis (DH) patients currently on a gluten-free diet with or without oats.

		Oats, *n* = 256 (82%)	No Oats, *n* = 56 (18%)	*p*-Value
Females, *n* (%)		125 (49)	26 (46)	0.745
Age at diagnosis, median (Q1–Q3), years		37 (27–50)	39 (24–48)	0.481
Year of diagnosis (dg), median (Q1–Q3)		1993 (1982–2002)	1988 (1982–2000)	0.184
	Dg < 1985, *n* (%)	74 (29)	18 (32)	
	Dg 1985–1999, *n* (%)	96 (38)	24 (43)	
	Dg 2000–2014, *n* (%)	86 (34)	14 (25)	
Duration of skin symptoms before diagnosis, median (Q1–Q3), months ^1^		11 (6–36)	10 (5–60)	0.671
Severity of skin symptoms ^2^ at diagnosis, *n* (%)^3^				0.076
	Mild	26/167 (16)	6/31 (19)	
	Moderate	90/167 (54)	10/31 (32)	
	Severe	51/167 (31)	15/31 (48)	
Gastrointestinal symptoms at diagnosis, *n* (%)		108/227 (48)	25/50 (50)	0.756
Small bowel histology at diagnosis, *n* (%)				0.530
	Normal	43/207 (21)	5/36 (14)	
	Partial villous atrophy	78/207 (37)	13/36 (36)	
	Subtotal/total villous atrophy	86/207 (42)	18/36 (50)	
Positive coeliac serology ^3^ at diagnosis, *n* (%)		121/164 (74)	19/31 (61)	0.156

Q1–Q3: Interquartile ranges. ^1^ Data available in 224 study patients (188 using oats, 36 not using oats). ^2^ Graded according to the presence of a few, several or many blisters, macular eruptions and erosions; ^3^ IgA-class anti-reticulin, endomysium or tissue transglutaminase antibody test.

**Table 2:** Long-term follow-up data of 312 dermatitis herpetiformis (DH) patients on a gluten-free diet (GFD) with or without of oats.

	Oats, *n* = 256 (82%)	No Oats, *n* = 56 (18%)	*p*-Value
Age, median (range), years	62 (18–96)	62 (32–85)	0.963
Duration of GFD, median (range), years	21 (1–47)	24 (2–41)	0.161
GFD adherence			0.229
Strict, no dietary lapses, *n* (%)	200/254 (79)	49/56 (88)	
Dietary lapses less than once a month, n (%)	36/254 (14)	6/56 (11)	
Dietary lapses once a month or more often, n (%)	18/254 (7)	1/56 (2)	
Skin symptoms, *n* (%)^1^	30/188 (16)	10/36 (28)	0.090
Dapsone treatment, *n* (%)^1^	8/188 (4)	5/36 (14)	0.040
Gastrointestinal symptoms, *n* (%)^1^	8/188 (4)	7/36 (19)	0.004
The total number of long-term illnesses, median (range)^1^	1 (0–12)	1 (0–9)	0.850
Coronary heart disease, *n* (%)	20 (8)	2 (4)	0.261
Cerebrovascular disease, *n* (%)	7 (3)	1 (2)	1.000
Osteoporosis or osteopenia, *n* (%)	15 (6)	3 (6)	1.000
Bone fractures, *n* (%)	49 (19)	12 (21)	0.696
Malignancy, *n* (%)	22 (9)	2 (4)	0.273
Number of prescription medications used, median (range) ^1^	2 (0–4)	2 (0–9)	0.510
Number of over-the-counter medications used, median (range) ^1^	1 (0–5)	1 (0–5)	0.769
Current smoking, *n* (%)^1^	18 (7)	9 (16)	0.032
Body mass index, kg/m^2^, median (range)^1^	25 (17–40)	26 (20–33)	0.242

^1^ Data available in 224 study patients (188 using oats, 36 not using oats).

**Table 3:** Median values and quartiles (Q_1_–Q_3_) for the Gastrointestinal Symptom Rating Scale (GSRS), Psychological General Well-Being (PGWB) and Dermatology Life Quality Index (DLQI) questionnaires’ total scores and sub-scores for the 312 dermatitis herpetiformis patients on a gluten-free diet with or without oats.

		Oats, *n* = 256 (82%)	No Oats, *n* = 56 (18%)	*p*-Value
		median (Q_1_–Q_3_)	median (Q_1_–Q_3_)	
GSRS scores ^1^				
	Total score	1.7 (1.3–2.2)	1.7 (1.4–2.2)	0.322
	Abdominal pain	1.7 (1.0–2.0)	1.7 (1.0–2.0)	0.722
	Reflux	1.0 (1.0–2.0)	1.0 (1.0–2.0)	0.483
	Diarrhoea	1.3 (1.0–2.0)	1.3 (1.0–2.7)	0.045
	Indigestion	2.0 (1.5–2.8)	2.0 (1.5–2.5)	0.931
	Constipation	1.3 (1.0–2.3)	1.7 (1.0–2.3)	0.570
PGWB scores ^2^				
	Total score	110 (99–117)	103 (94–118)	0.083
	Anxiety	26 (23–27)	25 (22–27)	0.364
	Depressed mood	17 (16–18)	17 (15–18)	0.181
	Positive well-being	18 (16–20)	17 (15–20)	0.266
	Self-control	16 (15–17)	15 (14–17)	0.145
	General health	14 (12–16)	13 (11–15)	0.020
	Vitality	19 (17–21)	18 (15–20)	0.025
DLQI score ^3, 4^		0 (0–0)	0 (0–1)	0.028

A higher score indicates ^1^ more severe symptoms, ^2^ better health-related well-being or ^3^ more impaired quality of life. ^4^ Data available in 224 study patients (188 using oats, 36 not using oats).
